# KCNQ1OT1 regulates the retinoblastoma cell proliferation, migration and SIRT1/JNK signaling pathway by targeting miR-124/SP1 axis

**DOI:** 10.1042/BSR20201626

**Published:** 2021-01-12

**Authors:** Haitao Zhang, Xin Yang, Yingying Xu, Haijun Li

**Affiliations:** Department of Ophthalmology, Henan Provincial People’s Hospital, Zhengzhou 450000, Henan, China

**Keywords:** KCNQ1OT1, miR-124, Retinoblastoma progression, Silent information regulator 1 (SIRT1)/c-Jun N-terminal kinase (JNK)

## Abstract

**Objective:** Long non-coding RNA (lncRNA) KCNQ1OT1 was reported to be tightly associated with tumorigenesis and progression of multiple cancers. However, the expression and biological functions of KCNQ1OT1 in retinoblastoma (RB) are still unknown. We aim to elucidate the potential function and underlying mechanism of KCNQ1OT1 in regulating the progression of RB. **Methods:** The levels of KCNQ1OT1 were assayed by real-time fluorescence quantitative polymerase chain reaction (RT-qPCR) analysis. The cell proliferation of RB cells (Y79 and WERI-Rb-1) were evaluated through Cell Counting Kit 8 (CCK-8) assay. Meanwhile, Y79 and WERI-Rb-1 cell apoptosis and cell cycle were assessed by Flow Cytometry analysis. Dual luciferase reporter assay were performed to illustrate the interaction between KCNQ1OT1, miR-124, and SP1. **Results:** We found that KCNQ1OT1 was up-regulated and miR-124 was down-regulated in RB tissues and cells. Moreover, knockdown of KCNQ1OT1 reduced the proliferation, migration, and cell cycle, as well as promoted cell apoptosis of Y79 and WERI-Rb-1 cells. Western blot analysis consistently proved cell cycle and apoptosis related protein expression levels. More importantly, KCNQ1OT1 was a sponge of microRNA (miR)-124. MiR-124 inhibition strongly reversed the effect on cell proliferation, cycle arrest, and apoptosis by KCNQ1OT1 knockdown mediation. In addition, KCNQ1OT1 regulated expression of SP1, a direct target of miR-124 in RB. On the other hand, miR-124 inhibitor abrogated the active effect of KCNQ1OT1 silencing on silent information regulator 1 (SIRT1)/c-Jun N-terminal kinase (JNK) signaling pathway. The function of KCNQ1OT1 was verified *in vivo*. **Conclusions:** These findings implied that KCNQ1OT1 silencing inhibited RB progression and activated SIRT1/JNK signaling pathway partially by modulating the miR-124/SP1 axis.

## Introduction

Retinoblastoma (RB) is the most common intraocular malignancy in infants and young children, which seriously harms children’s vision and life. Approximately 95% of children with RB develop the disease before the age of 5. The global incidence of RB is 1:15000 to 1:18000 [1]. In China, there are more than 1100 new cases each year [2]. Recently, with the advancement of RB early diagnosis and treatment, the survival rate of RB patients has improved significantly. However, RB is prone to metastasis and resistant to chemotherapy/radiotherapy, which increases the risk of blindness and death. Therefore, understanding the molecular mechanism of RB occurrence and development is crucial for the molecular targeted therapy for RB.

Long non-coding RNAs (LncRNAs) refer to non-coding RNAs more than 200 nucleotides. LncRNAs play a key role in several biological processes, such as cell cycle operation and embryonic development via regulating gene expression. In particular, lncRNAs are closely related to the occurrence and development of tumor, and have obvious abnormal expression in various tumor tissues and precancerous lesion tissues [3,4]. Related studies have found that many lncRNAs were abnormally expressed in RB, such as H19 [5], MALAT1 [6], and BANCR [7]. LncRNA KCNQ1OT1, a well-established lncRNA acts as an oncogene, was found to be highly expressed in many malignant tumors, such as non-small-cell lung carcinoma (NSCLC) [8], colon cancer [9], colorectal cancer [10], and so on. Nonetheless, the expression and biological effect of KCNQ1OT1 in RB remain to be investigated.

It is well known that lncRNA, microRNA (miRNA), and mRNA interactions are related to the formation of competing endogenous RNA (ceRNA) [11]. Emerging researches suggested that KCNQ1OT1 was involved in the occurrence, metastasis, and drug resistance of tumor via acting as a sponge for miRNAs, such as miR-217, miR-212-3, and miR-504 [10,12,13]. Interestingly, a previous study indicated that miR-124 was down-regulated in RB tissues and cell lines [14]. And miR-124/signal transducer and activator of transcription 3 (STAT3) axis partially reversed the lncRNA XIST-mediated cell proliferation, cell cycle arrest, and cell apoptosis in RB cells [14]. In the early stage of this experiment, we predicted that miR-124 had the same binding site with KCNQ1OT1 using bioinformatics software. This finding indicated that KCNQ1OT1/miR-124 might have potential function in the development of RB.

In the present study, we intended to uncover the function and mechanism of KCNQ1OT1 on proliferation and migration of RB. Specifically, we will show that KCNQ1OT1 and miR-124 mediate RB cell progression possibly via regulating SP1 expression and silent information regulator 1 (SIRT1)/c-Jun N-terminal kinase (JNK) signaling, which are strongly associated with tumor cell proliferation, apoptosis, and migration [15–17].

## Materials and methods

### Tissue sample collection

Three freshly frozen tissue samples of RB and their adjacent normal tissues were obtained from Henan Provincial People’s Hospital (Henan, China). All tissue samples were harvested at surgery, immediately frozen in liquid nitrogen, and stored at −80°C until RNA extraction. None of the patients received chemotherapy or local radiotherapy before surgery. Thise present study was approved by the Independent Ethics Committee (IEC) of Henan Provincial People’s Hospital (Henan, China, Ethics Approval Number HNEECA-2020-06). Each patient or their parents were informed and their written consent was acquired.

### Mice

Twelve female nude mice (4-week-old) were purchased from Dashuo Animal Experiment Co., Ltd. (Chengdu, Sichuan) and raised in the West China Animal Experiment Center of Sichuan University. The mice were kept in cages (four animals/cage) in a ventilated room. The feeding environment was 24 ± 1°C, relative humidity 55 ± 5%, and light/darkness for 12-h circulation. The mice were allowed to eat and drink freely. The present study was approved by the Sichuan University Ethics Committee (Ethics Approval Number HNEECA-2020-06).

### Construction of nude mice implanted tumor model

Twelve nude mice were randomly divided into two groups (*n*=6/group), namely shRNA-NC group and shRNA-KCNQ1OT1 group. The PBS suspended KCNQ1OT1 stable low-expression Y79 cells and the negative control cells (4 × 10^6^ cells/100 μl) were subcutaneously injected into nude mice (total injection of 2 × 10^6^ cells). Then, the nude mice were fed normally and observed the inoculation site for leaks. Observation indicators: weight, diet, mental, and tumor morphology were daily recorded. The diameter of the tumor was measured with a Vernier caliper. Four weeks after the successful establishment of the model, pentobarbital sodium (Merck, Germany, 20160411; 50 mg/kg) was injected intraperitoneally to anesthetize the nude mice. After tumor dissection, the experiment was terminated and all mice were killed by CO_2_. The tumor tissue was removed. Tumor volume calculation formula: V = 1/2 × L × D^2^ (L represents the largest diameter measured, D represents the smallest diameter measured).

### Cell culture

The human retinal pigment epithelial cell line (hTERT RPE-1) and the human RB cell lines (Y79 and WERI-Rb-1) were obtained from the American Type Culture Collection (ATCC). The cells were maintained in Roswell Park Memorial Institute-1640 (RPMI-1640, Sigma, St. Louis, MO, U.S.A.) medium supplemented with 10% Fetal bovine serum (FBS, HyClone Laboratories, Inc., South Logan, UT, U.S.A.), and 1% penicillin–streptomycin (Gibco, Rockville, MD, U.S.A.) in a humidified incubator with 5% CO_2_ and 95% air.

### Real-time fluorescence quantitative polymerase chain reaction

Total RNA were isolated using TRIzol® reagent (Takara Biotechnology, Dalian, China). The reverse transcription reaction condition was as follows: 95°C for 2 min; 40 cycles of 94°C for 20 s, 58°C for 20 s, and 72°C for 20 s. The sequences of specific miRNA RT primers (Invitrogen, Carlsbad, CA, U.S.A.) were as follows: KCNQ1OT1 forward, 5′- TGC AGA AGA CAG GAC ACT GG-3′ and reverse, 5′-TT TGG TGG GAA AGG ACA GA-3′; miR-124 forward, 5′- GCG CTA AGG CAC GCG GT-3′ and reverse, 5′-CAG TGC AGG GTC CGA GGT-3′; SP1 forward, 5′-GGC TCG GGG GAT CCT GGC-3′ and reverse, 5′-TAT GGC CCA TAT GTC TCT G-3′; GAPDH forward, 5′-CCA AGG TCA TCC ATG ACA AC-3′ and reverse, 5′-TGT CAT ACC AGG AAA TGA GC-3′. The relative gene expression level was determined using the 2^−ΔΔ*C*t^ method from ABI software, Foster City, CA.

### Western blot analysis

Total protein was separated by 10% SDS polyacrylamide gel electrophoresis and then transferred to nitrocellulose membranes (Millipore, Boston, MA, U.S.A.). The membranes were blocked with 5% nonfat milk and incubated with corresponding protein antibodies or a rabbit anti-β-actin monoclonal antibody. Then, the membranes were subsequently incubated with an HRP Goat anti-Rabbit IgG (1:20000; Boster, Wuhan, China; BA1054). The proteins were detected by the film scanner (Microtek, Shanghai, China) and β-actin was used as an internal control. The net optical density was analyzed with the Gel Image processing system (ImagePro Plus 6.0). Primary antibodies used were as follows: CDK2 (Abcam, Cambridge, U.K.; cat. no. ab32147, 1:2000), Cyclin D1 (Abcam, Cambridge, U.K.; cat. no. ab134175, 1:10000), MMP2 (CST, Boston, MA, U.S.A.; cat. no. 40994, 1:1000), MMP9 (CST, Boston, MA, U.S.A.; cat. no. 13667, 1:1000), E-Cadherin (CST, Boston, MA, U.S.A.; cat. no. 3159), N-Cadherin (CST, Boston, MA, U.S.A.; cat. no. 13116), vimentin (CST, Boston, MA, U.S.A.; cat. no. 13116), SIRT1 (Abcam, Cambridge, U.K.; cat. no. ab220807, 1:2000), p-JNK (CST, Boston, MA,U.S.A.; cat. no. 9255, 1:2000), JNK (Abcam, Cambridge, U.K.; cat. no. ab76125, 1:2000), pro-caspase3 (Abcam, Cambridge, U.K.; cat. no. ab32499, 1:10000), cleaved-caspase3 (Abcam, Cambridge, U.K.; cat. no. ab32042, 1:500), and β-actin (CST, Boston, MA, U.S.A.; cat. no. 4970, 1:1000).

### Cell transfection

Small interfering RNA (siRNA) targeting KCNQ1OT1 and the corresponding negative control were designed by GeneChem (Shanghai, China). The miR-124 mimics, inhibitors, and corresponding negative controls were purchased from GeneChem. Oligonucleotides were transfected into the cell lines using Lipofectamine 3000 (Thermo Fisher Scientific) according to the manufacturer’s protocol.

### Dual luciferase reporter assay

Sta,rbase (http://starbase.sysu.edu.cn/) predicted the binding region of KCNQ1OT1 and miR-124. The KCNQ1OT1 3′ UTR sequence was cloned to pSI-Check2 (Hanbio Biotechnology) to form the vector pSI-Check2-KCNQ1OT1-wild-type (pGL3-KCNQ1OT1-wt). And KCNQ1OT1 3′ UTR mutant sequence was also cloned to pSI-Check2 to form the vector pSI-Check2-KCNQ1OT1-mut-type (pGL3-KCNQ1OT1-mut). 293T cells were cotransfected with NC mimics/miR-124 mimics and pGL3-KCNQ1OT1-wt/pGL3-KCNQ1OT1-mut. Finally, the luciferase activities were analyzed using a dual-luciferase reporter assay system (Promega, Madison, Wisconsin, WI, U.S.A.).

### Cell vitality assay

The cell vitality of human RB cell lines Y79 and WERI-Rb-1 were measured using the Cell Counting Kit 8 (CCK-8, Dojindo, Cat.No. CK04) according to the manufacturer’s instructions. The cell vitality was measured using enzyme-linked immune monitor at 450 nm.

### Cell migration assay

Cell migration was determined by Transwell plates (Corning, NY) that were uncoated or coated with 50 μl of Matrigel (BD, NJ). A total of 5 × 10^4^ Y79 and WERI-Rb-1 cells were resuspended in 250 μl of serum-free medium and added to the upper chamber, while the lower chamber was filled with 500 μl of complete medium. Then, the chambers were incubated for 48 h (5% CO_2_, 37°C). Cells in the upper chambers were fixed with methanol and stained with 0.1% Crystal Violet. Migrated cells were counted in three randomly selected microscopic fields.

### Cell apoptosis and cell cycle analysis

Y79 and WERI-Rb-1 cells from corresponding group were incubated in a 24-well plate for 48 h. A total of 500 μl of the cell suspension was mixed with 2 μl of Annexin (1 mg/ml) and 2 μl of propidium iodide (1 mg/ml) (Abcam, Cambridge, U.K.; ab14083). Then, the cells were analyzed using a flow cytometer. Annexin-positive and propidium iodide-positive cells were considered apoptotic. Cell cycle analysis was performed using propidium iodide (Beyotime, ShangHai, China; C1052) with the BD FACSCalibur Flow Cytometry System (BD Pharmingen).

### Statistical analysis

The data were represented as means ± standard deviation (SD) and each experiment was performed in triplicate in the present study. ANOVA with post hoc test of means and Student’s unpaired *t* test were used to assess statistical significance, which was done using SPSS 22.0 software (SPSS, Inc., Chicago, IL, U.S.A.) and GraphPad Prism 7 (GraphPad Inc., San Diego, CA, U.S.A.). *P*-value <0.05 was considered statistically significant.

## Results

### LncRNA KCNQ1OT1 was up-regulated in RB tissues in RB cells

**Figure 1:**
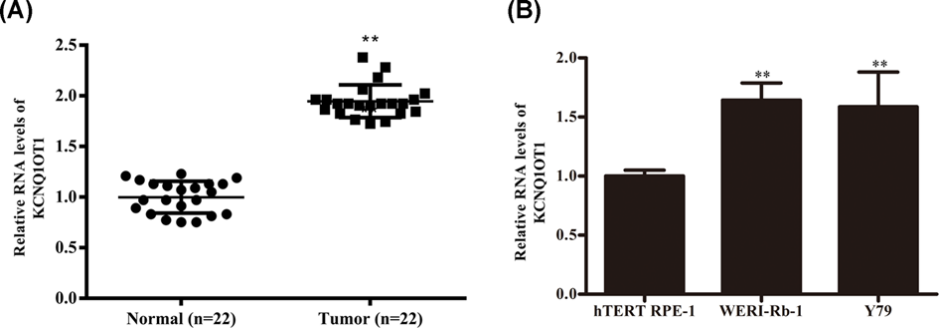
LncRNA KCNQ1OT1 was up-regulated in RB tissues in RB cells (**A**) The expression of KCNQ1OT1 in human RB tissues (*n*=3) and adjacent normal tissues (*n*=3) were compared by RT-qPCR analysis. (**B**) The relative RNA levels of KCNQ1OT1 in human retinal pigment epithelial cell line (hTERT RPE-1) and RB cell line (Y79 and WERI-Rb-1) were detected by RT-qPCR analysis. **P<0.01 (vs. NC-siRNA).

Real-time fluorescence quantitative polymerase chain reaction (RT-qPCR) was introduced to analyze the level of KCNQ1OT1 in RB tissues in RB cells. Compared with the adjacent normal tissues, KCNQ1OT1 was up-regulated in RB tissues (Figure 1A). Identically, KCNQ1OT1 was highly expressed in Y79 and WERI-Rb-1 cells (Figure 1B).

### Silencing of KCNQ1OT1 inhibited cell proliferation, migration, and promoted cell apoptosis of RB cells

**Figure 2:**
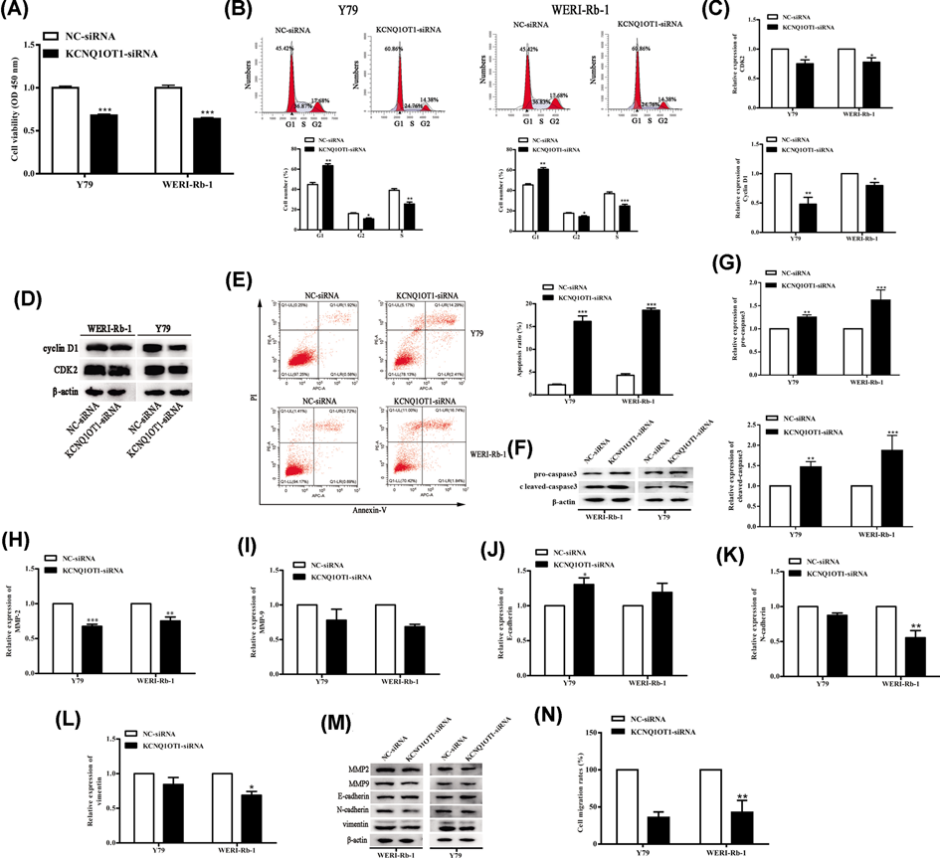
Silencing of KCNQ1OT1 inhibited cell proliferation, migration, and promoted cell apoptosis of RB cells Y79 and WERI-Rb-1 cells were transfected with NC-siRNA or siRNA-KCNQ1OT1 for 48 h. (**A**) The proliferation of Y79 and WERI-Rb-1 cells was detected by CCK-8 assay. (**B,E**) The cell apoptosis and cell cycle of Y79 and WERI-Rb-1 cells were tested using Flow Cytometry. (**C,D,F**–**M**) The expression of CDK2, cyclinD1, pro-caspase3, cleaved-caspase3, MMP2, MMP9, N-Cadherin, vimentin, and cadherin expression in Y79 and WERI-Rb-1 cells were assayed using Western blot analysis. β-actin is a loading control. (**N**) The migration of Y79 and WERI-Rb-1 cells was tested using Transwell assay. *P<0.05 (vs. NC-siRNA), **P<0.01 (vs. NC-siRNA), ***P<0.001 (vs. NC-siRNA).

Then, we identified the functions of KCNQ1OT1 on the viability of RB cells. Y79 and WERI-Rb-1 cells were transfected with either NC-siRNA or KCNQ1OT1-siRNA. CCK-8 assay revealed that KCNQ1OT1 silencing inhibited cell viability in Y79 and WERI-Rb-1 cells (Figure 2A). The transfection of KCNQ1OT1-siRNA successfully reduced cell cycle arrest (Figure 2B). Meanwhile, Western blot analysis evidenced that KCNQ1OT1 silencing significantly reduced CDK2 and cyclin D1 expression in Y79 and WERI-Rb-1 cells (Figure 2C,D). In addition, the apoptosis level of Y79 and WERI-Rb-1 cells was promoted by KCNQ1OT1-siRNA (Figure 2E). And elevated pro-caspase3 and cleaved-caspase3 expression levels validated this result (Figure 2F,G). The expression of migration-related proteins MMP2, MMP9, N-Cadherin, and vimentin were partly reduced, as well as E-cadherin expression was induced in KCNQ1OT1 siRNA transfected Y79 and/or WERI-Rb-1 cells (Figure 2H–M). Moreover, KCNQ1OT1 knockdown obviously inhibited cell migration of Y79 and WERI-Rb-1 cells (Figure 2N). Taken together, these data demonstrated that down-regulation of KCNQ1OT1 suppressed the proliferation and migration, and promoted the apoptosis of Y79 and WERI-Rb-1 cells.

### KCNQ1OT1 positively regulated SP1 expression through miR-124

**Figure 3:**
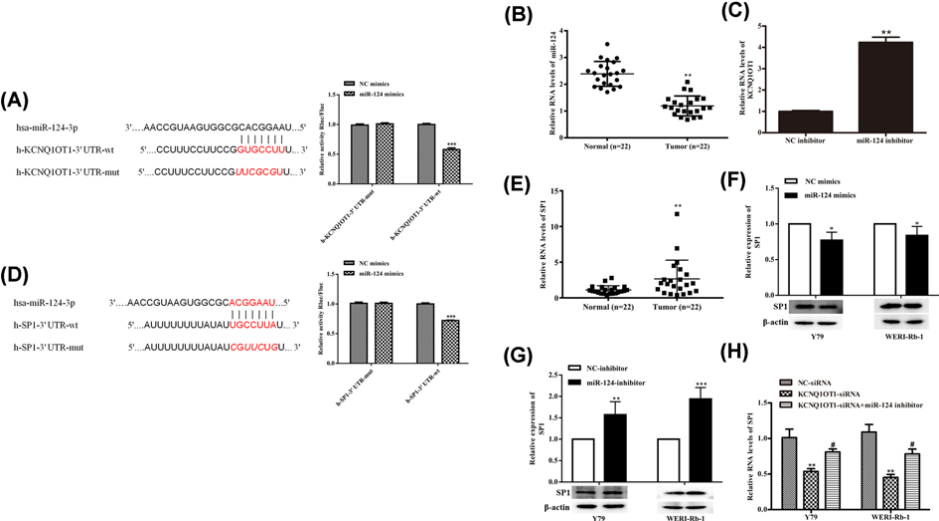
KCNQ1OT1 positively regulated SP1 expression through miR-124 (**A**) The binding regions of miR-124 and KCNQ1OT1 3′ UTR sequences are flagged use red. Dual luciferase reporter assay was used to assay the interaction of miR-124 and KCNQ1OT1 3′ UTR sequences. (**B**) The RNA levels of miR-124 in human RB tissues (*n*=3) and adjacent normal tissues (*n*=3) were tested by RT-qPCR analysis. (**C**) The expression of KCNQ1OT1 was tested by RT-qPCR analysis. (**D**) The binding regions of miR-124 and SP1 3′ UTR sequences are flagged use red. Dual luciferase reporter assay was used to assay the interaction of miR-124 and SP1 3′ UTR sequences. (**F,G**) The expression of SP1 was assayed using Western blot analysis. β-actin is a loading control. (**E,H**) The RNA level of SP1 was measured by RT-qPCR analysis. *P<0.05 (vs. Normal/NC mimics), **P<0.01 (vs. NC inhibitor), ***P<0.001 (vs. NC mimics), ^#^*P*<0.05 (vs. KCNQ1OT1 siRNA).

The binding regions of KCNQ1OT1 3′UTR sequence and miR-124 were predicted using Starbase (http://starbase.sysu.edu.cn/, Figure 3A). Of note, dual-luciferase reporter gene assay showed that the luciferase activity was significantly down-regulated in miR-124 mimics transfected 293T cells, confirming the target relationship between miR-124 and KCNQ1OT1 3′UTR sequence (Figure 3A). And RT-qPCR analysis showed that miR-124 level was down-regulated in RB tissues compared with the adjacent normal tissues (Figure 3B). We also found that miR-124 knockdown obviously increased KCNQ1OT1 expression (Figure 3C). The transcription factor SP1, which is a known regulator of the development of various cancers, including RB [18]. Consistent with previous data, we also found SP1 expression was increased in RB tissues (Figure 3E). The study has shown that SP1 is a critical target of miR-124 in vascular smooth muscle cells (VSMCs) [19] and mesenchymal stem cells [20]. Hence, we further evaluated the relationship between SP1 and miR-124. In our study, dual-luciferase assay revealed that miR-124 directly bound to the 3′ UTR of SP1 protein (Figure 3D). Meanwhile, miR-124 overexpression inhibited SP1 expression in Y79 and WERI-Rb-1 cells (Figure 3F). And miR-124 inhibitor significantly activated SP1 expression in protein level in Y79 and WERI-Rb-1 cells (Figure 3G). Notably, KCNQ1OT1 knockdown inhibited SP1 expression, which was supplemented by miR-124 inhibitor cotransfection (Figure 3H). Taken together, these results suggested that miR-124 is a target of KCNQ1OT1, and SP1 is a target of miR-124 in RB cells.

### MiR-124 knockdown abolished the effect of KCNQ1OT1 silencing on cell viability, migration, and apoptosis of RB cells

**Figure 4:**
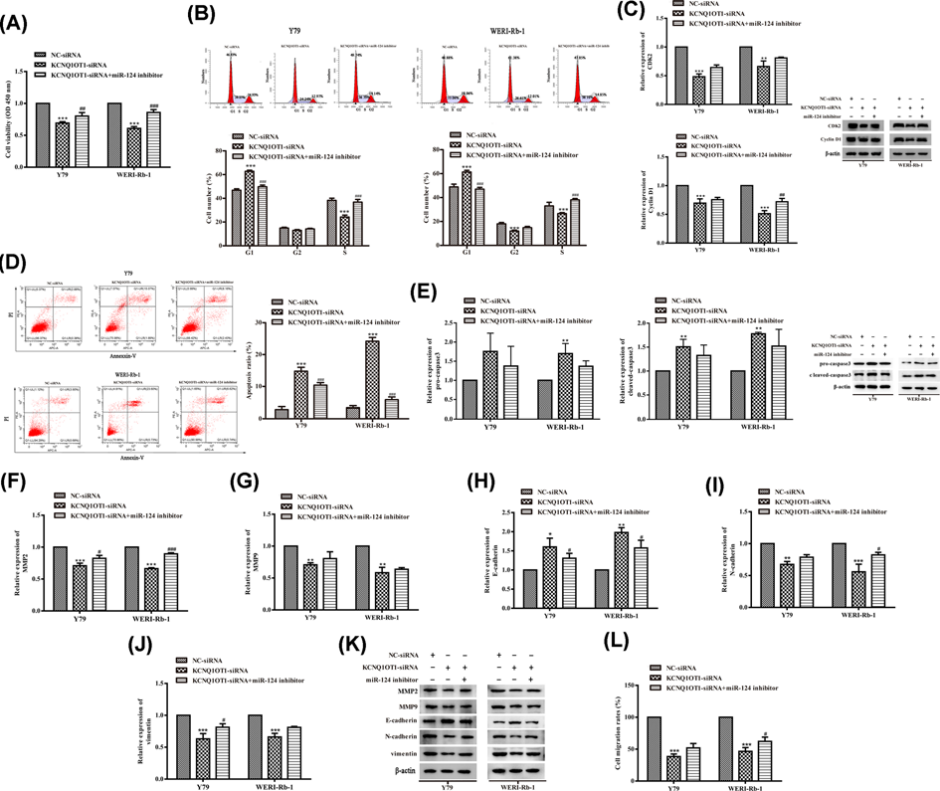
MiR-124 knockdown abolished the effect of KCNQ1OT1 silencing on cell viability, migration, and apoptosis of RB cells Y79 and WERI-Rb-1 cells were transfected with NC-siRNA, miR-124 inhibitor, or/and KCNQ1OT1 siRNA. (**A**) The cell viability of Y79 and WERI-Rb-1 cells were tested by CCK-8 assay. (**B**) The cell cycle of Y79 and WERI-Rb-1 cells were tested using Flow Cytometry. (**C**) The expression of CDK2 and cyclinD1 in Y79 and WERI-Rb-1 cells were assayed using Western blot analysis. β-actin is a loading control. (**D**) The cell apoptosis of Y79 and WERI-Rb-1 cells were tested using Flow Cytometry. (**E**) The expression of pro-caspase3 and cleaved-caspase3 in Y79 and WERI-Rb-1 cells were assayed using Western blot analysis. β-actin is a loading control. (**F**–**K**) The expression of MMP2, MMP9, N-Cadherin, vimentin, and cadherin expression in Y79 and WERI-Rb-1 cells were assayed using Western blot analysis. β-actin is a loading control. (**L**) The migration of Y79 and WERI-Rb-1 cells were measured using Transwell assay. *P<0.05 (vs. NC-siRNA), **P<0.01 (vs. NC-siRNA), ***P<0.001 (vs. NC-siRNA), ^#^*P*<0.05 (vs. KCNQ1OT1 siRNA), ^##^*P*<0.01 (vs. KCNQ1OT1 siRNA), ^###^*P*<0.001 (vs. KCNQ1OT1 siRNA).

Then, Y79 and WERI-Rb-1 cells were cotransfected with miR-124 inhibitor and KCNQ1OT1 siRNA. As expected, silencing of KCNQ1OT1 decreased cell viability of Y79 and WERI-Rb-1 cells, which were activated by miR-124 inhibitor cotransfection (Figure 4A). Meanwhile, miR-124 inhibitor significantly enhanced cell cycle arrest at the S-phase of Y79 and WERI-Rb-1 cells (Figure 4B). And the decreased CDK2 and cyclin D1 expression caused by KCNQ1OT1 siRNA were replenished in miR-124 inhibitor cotransfected Y79 and WERI-Rb-1 cells (Figure 4C). In addition, miR-124 inhibitor + KCNQ1OT siRNA inhibited Y79 and WERI-Rb-1 cell apoptosis (Figure 4D). And the increase in pro-caspase3 and cleaved-caspase3 expression levels were slightly reversed by miR-124 silencing, although the data were not significant (Figure 4E). Concurrently, our results also displayed that KCNQ1OT1 siRNA receded MMP2, MMP9, N-Cadherin, and vimentin expression as well as strengthened E-cadherin expression both in Y79 and WERI-Rb-1 cells, which were partly counteracted by miR-124 inhibitor (Figure 4F–K). In addition, miR-124 inhibitor + KCNQ1OT siRNA also promoted Y79 and WERI-Rb-1 cell migration (Figure 4L). These results implied that KCNQ1OT1 exerted its biological function in RB cells by partially regulating miR-124/SP1 axis. Collectively, these results indicated that KCNQ1OT regulates the proliferation, migration, and apoptosis of RB cells via negatively modulating miR-124 expression.

### MiR-124 was involved in the KCNQ1OT1-mediated SIRT1/JNK signaling pathway of RB cells

**Figure 5:**
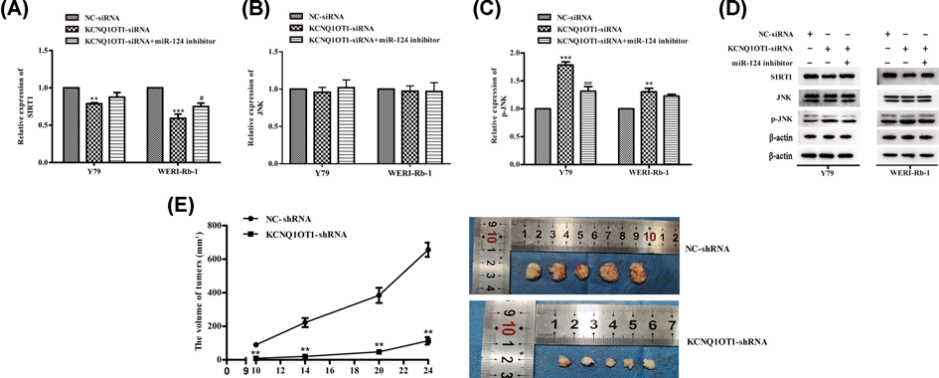
MiR-124 was involved in the KCNQ1OT1-mediated SIRT1/JNK signaling pathway of RB cells and KCNQ1OT1 silencing suppresses NSCLC development *in vivo* Y79 and WERI-Rb-1 cells were transfected with NC-siRNA, miR-124 inhibitor, or/and KCNQ1OT1 siRNA. (**A–D**) The expression of SIRT1, JNK, p-JNK were assayed using Western blot analysis. β-actin is a loading control. (**E**) Compared with the shRNA-NC group, shRNA-KCNQ1OT1 inhibited the tumor volume (bar = 10 mm). **P<0.01 (vs. NC-siRNA/shRNA), ***P<0.001 (vs. NC-siRNA), ^#^*P*<0.05 (vs. KCNQ1OT1 siRNA), ^###^*P*<0.001 (vs. KCNQ1OT1 siRNA).

It has been reported that histone deacetylase SIRT1 which can eliminate the generation of ROS in cancer cells [21,22]. On the other hand, JNK is a molecular linkage between oxidative stress and cell apoptosis [23]. Thus, we assessed if KCNQ1OT1 regulates SIRT1/JNK signaling pathway both in Y79 and WERI-Rb-1 cells. Western blot analysis found that miR-124 inhibitor reversed the effect of KCNQ1OT1 depletion on SIRT1 deactivation and JNK phosphorylation (Figure 5A–D), indicating that KCNQ1OT1 may induce SIRT1/JNK signaling availability via miR-124/SP1 axis.

### Down-regulation of KCNQ1OT1 suppresses the RB growth *in vivo*

We further evaluated the role and mechanism of KCNQ1OT1 *in vivo*. We subcutaneously injected Y79 cells with shRNA-KCNQ1OT1 or negative regulator vector into the nude mice. After 28 days, the mice were killed, and the tumors were examined. The data indicated that down-regulation of KCNQ1OT1 remarkably suppressed tumor volumes and weight compared with the control groups (Figure 5E).

## Discussion

The current studies revealed that several lncRNAs were involved in the occurrence, progression, and migration of RB. For example, lncRNA PLAC2 induced RB cell apoptosis via regulating PTEN expression [24]. Knockdown of lncRNA XIST repressed the proliferation and invasion of RB cells by miR-140-5p/SOX4 signaling [25]. And lncRNA TP73-AS1 activated TFAP2B-mediated cell proliferation, metastasis, and invasion in RB via decoying miRNA-874-3p [26]. Accumulating evidences displayed that KCNQ1OT1 was a promising cancer-related lncRNA. Dramatically, KCNQ1OT1 was up-regulated in tumor tissues and cells of colon cancer [9], osteosarcoma [27], gastric cancer [28], and NSCLC [29]. In our results, KCNQ1OT1 was found to highly express in RB tissues and RB cells. It has been reported that knockdown of KCNQ1OT1 in cancer cell lines strongly inhibited cell migration and proliferation. Thus, we further studied the effect of KCNQ1OT1 on RB cell viability. KCNQ1OT1 silencing suppressed proliferation and cell cycle arrest at the S-phase, as well as promoted apoptosis. On the other hand, it has been reported that KCNQ1OT1 could be used as ceRNA in combination with miR-7-5p/miR-27b-3p/miR-4458 to regulate tumor progression [27,30,31]. Therefore, we used the software StarBase2.0 (http://starbase.sysu.edu.cn) to search for miRNAs that could target KCNQ1OT1. Among miRNAs, miR-124 was predicted as a target miRNA of KCNQ1OT1 based on its roles in RB. Dual luciferase reporter assay proved that KCNQ1OT1 interacted with miRNA-24 in RB cells. We also found that miR-124 overexpression obviously decrease KCNQ1OT1 expression in RB cells.

miRNAs are involved in the regulation of cell proliferation, migration, and other processes. In addition, miRNAs play an important role in the occurrence and development of major human diseases, such as hypertension, diabetes, and tumor [32]. There is considerable evidence to suggest that miR-124 expression is closely related to the occurrence and development of cancers. Of note, several studies proved that miR-124 functioned as tumor suppressor in human RB. MiR-124 significantly inhibited proliferation and invasion of human RB cells by targeting STAT3 [33]. Similarly, miR-124 also inhibited MALAT1-induced RB cell autophagy through regulation of Syntaxin 17 (STX17) expression [34]. In the present study, we proved that miR-124 was down-regulated in RB tissues. And rescue experiments confirmed that the inhibition of miR-124 could reverse the effect of KCNQ1OT1 on RB cell proliferation, cycle arrest, apoptosis, and related protein activities.

LncRNA could function as a ceRNA to sponge the miRNAs, thereby affecting the expression of the target genes of miRNAs [35]. We found that miR-124 exerted its suppressor role in RB by targeting SP1. SP1, a member of the SP/Kruppel-like factor superfamily (Sp/KLF family) of transcription factors, has been reported to be highly expressed in pancreatic cancer [36], colorectal cancer [37], gastric cancer [38], and lung cancer [39]. Furthermore, SP1 participated in tumor progression via regulating multiple downstream genes, such as vimentin, cadherin, E-cadherin, and CDK2 [16,40]. Our results demonstrated that KCNQ1OT1 knockdown suppressed SP1 expression, which was supplemented by miR-124 inhibitor cotransfection, implying that there is a specific KCNQ1OT1/miR-124/SP1 axis regulating RB progression.

SIRT1, a class III histone deacetylase, could increase the risk of cancer [41]. The main function of SIRT1 is to deacetylate certain proteins that play a key role in gene expression, thereby regulating its downstream-related signaling pathways and accelerating the cell cycle, reducing apoptosis, and causing rapid proliferation of tumor cells. Of note, SIRT1 overexpression stimulates the cellular level of superoxide dismutase (SOD), which eliminates the generation of apoptosis promoter ROS [42–44]. Meanwhile, JNK phosphorylation has been demonstrated to be crucial for oxidative stress and ROS-dependent tumor cell apoptosis [45,46]. More importantly, miR-124 overexpression suppressed the expression of SIRT1 and thus promoted the phosphorylation of JNK and finally sensitized cisplatin-induced cytotoxicity against CD133^+^ hepatocellular carcinoma cells [47]. And in NSCLC cells, miR-140 resensitized cisplatin-resistant cells to cisplatin treatment by targeting SIRT1/ROS/JNK pathway [48]. In the present study, we found that KCNQ1OT1 knockdown promoted SIRT1/JNK signaling pathway both in Y79 and WERI-Rb-1 cells, while miR-124 inhibitor reversed this effect.

Taken together, our study confirmed that KCNQ1OT1 knockdown suppressed cell proliferation, migration, as well as promoted to cell apoptosis and SIRT1/JNK signaling by altering the miR-124/SP1 axis in RB. Our study may serve as novel potential biomarkers for targeted therapy of RB.

## Abbreviations

CCK-8: cell counting kit 8
CDK2: cyclin dependent kinase 2
ceRNA: competing endogenous RNA
GAPDH: glyceraldehyde-3-phosphate dehydrogenase
JNK: c-Jun N-terminal kinase
lncRNA: long non-coding RNA
MALAT1: metastasis associated lung adenocarcinoma transcript 1
miRNA: microRNA
MMP: matrix Metallopeptidase
NSCLC: non-small-cell lung carcinoma
RB: retinoblastoma
ROS: reactive oxygen species
RT-qPCR: real-time fluorescence quantitative polymerase chain reaction
siRNA: small interfering RNA
SIRT1: silent information regulator 1
STAT3: signal transducer and activator of transcription 3
TFAP2B: transcription factor AP-2 beta
